# Low-Dose PET Imaging of Tumors in Lung and Liver Regions Using Internal Motion Estimation

**DOI:** 10.3390/diagnostics11112138

**Published:** 2021-11-18

**Authors:** Sang-Keun Woo, Byung-Chul Kim, Eun Kyoung Ryu, In Ok Ko, Yong Jin Lee

**Affiliations:** 1 Division of RI-Convergence Research, Korea Institute of Radiology and Medical Sciences, Seoul 01812, Korea; xikian@kirams.re.kr (B.-C.K.); inogi99@kirams.re.kr (I.O.K.); yjlee@kirams.re.kr (Y.J.L.); 2 Center of Magnetic Resonance Research, Korea Basic Science Institute, Ochang 28119, Korea; ekryu@kbsi.re.kr

**Keywords:** animal model, imaging, rat, radioisotope, respiratory organ

## Abstract

Motion estimation and compensation are necessary for improvement of tumor quantification analysis in positron emission tomography (PET) images. The aim of this study was to propose adaptive PET imaging with internal motion estimation and correction using regional artificial evaluation of tumors injected with low-dose and high-dose radiopharmaceuticals. In order to assess internal motion, molecular sieves imitating tumors were loaded with ^18^F and inserted into the lung and liver regions in rats. All models were classified into two groups, based on the injected radiopharmaceutical activity, to compare the effect of tumor intensity. The PET study was performed with injection of F-18 fluorodeoxyglucose (^18^F-FDG). Respiratory gating was carried out by external trigger device. Count, signal to noise ratio (SNR), contrast and full width at half maximum (FWHM) were measured in artificial tumors in gated images. Motion correction was executed by affine transformation with estimated internal motion data. Monitoring data were different from estimated motion. Contrast in the low-activity group was 3.57, 4.08 and 6.19, while in the high-activity group it was 10.01, 8.36 and 6.97 for static, 4 bin and 8 bin images, respectively. The results of the lung target in 4 bin and the liver target in 8 bin showed improvement in FWHM and contrast with sufficient SNR. After motion correction, FWHM was improved in both regions (lung: 24.56%, liver: 10.77%). Moreover, with the low dose of radiopharmaceuticals the PET image visualized specific accumulated radiopharmaceutical areas in the liver. Therefore, low activity in PET images should undergo motion correction before quantification analysis using PET data. We could improve quantitative tumor evaluation by considering organ region and tumor intensity.

## 1. Introduction

Positron emission tomography (PET) provides functional images, including biological information, using radiopharmaceuticals emitting positrons. PET system allows imaging of biochemical changes in tumors before morphological changes, unlike anatomical imaging diagnostic methods, such as computed tomography (CT) or magnetic resonance imaging (MRI). The small animal PET scanner is widely used in noninvasive molecular imaging research in the preclinical stage because of its high sensitivity and spatial resolution. However, when the injected radiopharmaceutical activity was low, the PET image quality was also quite low, and was insufficient for detecting specific areas in small animals. However, when performing the clinical study, high injected doses of radiopharmaceuticals were harmful to subjects.

Respiration and cardiac motion induce degradation of image quality and quantity by causing deficiency of count and blurring of lesions during PET acquisition [1]. Accordingly, motion correction is necessary for the improvement of quantitative tumor evaluation and for preventing decline in image quality while acquiring PET images [2]. In order to minimize these repercussions, various motion estimation methods measuring external motion have been introduced. These methods include the detection of pressure variations using pressure sensors, and optical motion tracking systems, such as POLARIS (Northern Digital, Inc., Waterloo, Canada) and the charge-coupled device (CCD) camera [3,4,5].

Various techniques have been researched to correct for motion due to the cardiac and respiratory cycle. Methods using image registration [1,6,7] and optical flow algorithms [8,9] have been applied to reconstructed images as post-processing stages. Among preprocessing methods, some approaches used system matrix in the image reconstruction period [10,11,12], and some used rigid or affine algorithms in sinogram correction [13,14]. All the above-mentioned methods require estimates of organ motion. Currently, the respiratory gating method is most commonly used for motion correction, and the electrocardiogram (ECG) gating method is used for cardiovascular disease study [14,15].

Previous studies have shown that the gated PET method using an external monitoring device provides motion-corrected images. When the number of gates increases, the acquired image becomes similar to the real shape; but the contrast in the image is reduced by noise increase due to loss of count [16]. The monitoring data do not accurately represent the real organ motion [17]. The lungs and liver are among the organs most influenced by respiration and heartbeat; therefore, motion compensation is important for tumors located near the thoracic abdomen [17].

In this study, we designed the lung and liver motion model imaging with dual cardiac-respiratory gating for regional tumor quantification [18]. We tried to ascertain acute internal motion in PET images with 2-deoxy-2-[^18^F] fluoro-D-glucose (^18^F-FDG) by insertion of artificial tumors containing radioactive substances [19]. The purpose of this study was the improvement of quantitative tumor evaluation, depending on the target organ region and tumor intensity, through internal motion estimation and correction using thoracic artificial tumors.

## 2. Materials and Methods

### 2.1. Motion Model Preparation

All protocols of this study were approved by the Institutional Animal Care and Use Committee of the Korea Institute of Radiological and Medical Science (KIRAMS 2012-13). This study was performed in accordance with the guidelines of the KIRAMS and the Guide for the Care and use of Laboratory Animals [6]. Eight female Sprague-Dawley (SD) strain rats, aged 6 weeks and weighing approximately 300 g, were employed in these experiments. They were purchased from Harlan Laboratories (Indianapolis, IN, USA). They were quarantined 6 days before surgical procedure. All rats were considered to be in good health on the basis of physical examinations. The rats were housed in a facility approved by KIRAMS and were fed a standard diet. Rats were anesthetized by isoflurane inhalation anesthesia (2% mixed with 100% oxygen by the endotracheal catheter; Foran^®^, Choongwae Pharma Co., Suwon, Korea) [20]. In order to supply oxygen during open chest surgery, we disinfected the neck region of the rats by povidone—iodine and ethanol. The dedicated small animal ventilator (DJI-101, Daejong Instrument Industry Co., Seoul, Korea) was connected and the catheter was inserted. Temperature was achieved at 30 °C using a plastic pad with a water-filled chamber and an infrared ramp during injection and uptake time.

**Figure 1:**
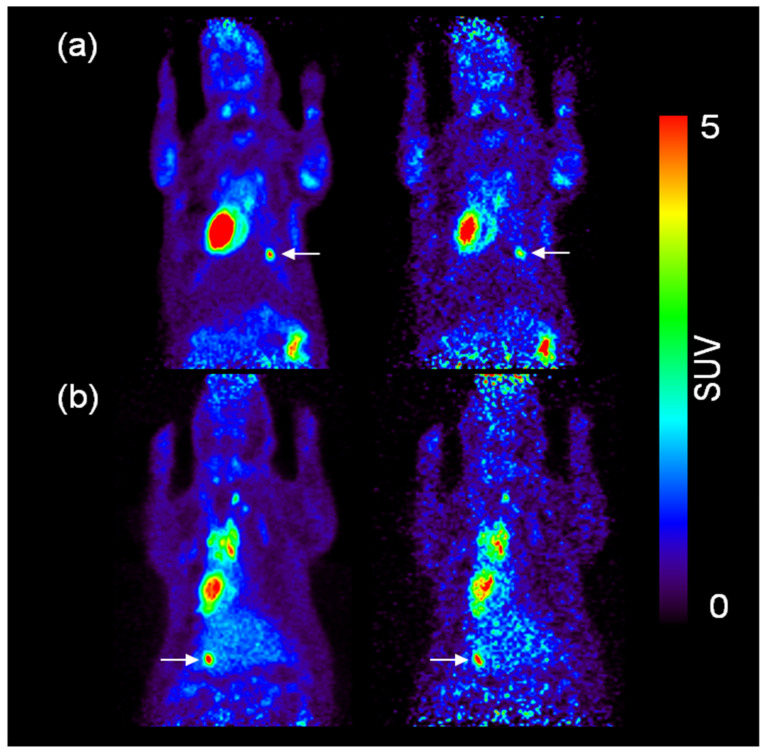
The results of motion correction PET image. (**a**) The left side image represents no motion correction, and the right side is a motion correction image of the lung. (**b**) The left side of the PET image represents no motion correction in liver, and right side represents a motion correction image.

Rats were divided into 2 groups according to the region planted with the molecular sieve containing radioactive material; one was the lung region group and the other the liver region group. As shown in Figure 1a, the planting surgeries in the lung region were performed inside the opened thorax region. Skin and intercostalis muscle were incised at the right 8th intercostal region, then the incised thoracic wall was expanded using surgical retractor. The molecular sieve was attached on the sternal surface of the right caudal lung lobe. In the liver region group, skin and abdominal muscles were incised at the left cranial abdominal region following the rib line and expanded by retractor. The molecular sieve was inserted into the left medial lobe of the liver. After attachment and insertion of the molecular sieve, incision sites were closed with 4-0 silk suture. The experimental conditions, including warming and anesthesia, were maintained during the entire study period.

Molecular sieves were immersed in 70% ethanol for one whole day before study and were allowed to absorb ^18^F on the verge of surgery. We adjusted the absorbed activity level of molecular sieves, and thus classified them into two groups: the high-activity group (about 0.67 MBq) and the low-activity group (about 0.37 MBq). All the molecular sieves were coated with thermoreversible gel (Pluronic^®^ 127F hydrogel) for quantitative estimation [21]. We excluded the models with poor vital conditions, such as respiratory, anesthesia, suture, and ^18^F-FDG uptake, from the experimental group.

### 2.2. PET Image Acquisition

All PET images in each region were acquired on a dedicated small animal PET scanner (Inveon^TM^, Siemens Preclinical Solutions, Knoxville, TN, USA), as shown in Figure 1b, after radioactive molecular sieve insertion under anesthesia maintenance. This PET scanner had Lutetium oxyorthosilicate (LSO) with 1.6 mm × 1.6 mm detector pixel spacing. The rats were injected with ^18^F-FDG (37 MBq in 0.2 mL) via the tail vein. For sufficient FDG distribution in the body, a 60 min uptake period was required following the injection. PET images were obtained as list-mode data for 20 min. Breathing signals were collected from pneumatic sensors attached to the thorax of the rat [22]. Cardiac signals were obtained from ECG by standard limb lead method. These signals were converted into trigger signals by the external motion monitoring system (BioVet, m2m Imag. Corp., Newark, NJ, USA). The threshold value of the trigger event was determined at systole by R-wave from ECG and inhale state of respiration cycle. The trigger signals simultaneously reflected the motion of the heart and breathing using the dual-trigger method [23]. Each line of response in the list-mode data was converted into sinogram gated various frames (2~16 bin) by a respiratory trigger event and an ECG trigger event, simultaneously. The acquired emission data were reconstructed using Fourier rebinning and ordered subsets expectation maximization 2D (OSEM 2D) algorithm with 4 iterations.

### 2.3. MRI Data Acquisition

MRI studies were performed using a 3T clinical MRI system (Magnetom Tim Trio, Siemens Medical Solutions, Erlangen, Bavaria, Germany) with a human wrist coil. Coronal 2D MR images were acquired using a T1-FLASH sequence with respiratory triggering and integrated parallel acquisition techniques (iPAT). The parameters were as follows: repetition time = 65.25 ms, echo time = 3.58 ms, flip angle = 12°, slice thickness = 2.5 mm, filter = distortion correction, phase oversampling = 10%, and field of view = 150 mm.

Coronal 3D MRI were acquired using a T1-VIBE sequence with generalized autocalibrating partially parallel acquisitions (GRAPPA). The parameters were as follows: repetition time = 5.67 ms, echo time = 1.42 ms, flip angle = 10°, slice thickness = 2.11 mm, filter = elliptical filter, phase oversampling = 1%, field of view = 200 mm, and slices per slab = 12.

A tagged MRI study was performed using a 4.7 T MRI system (BioSpec, Bruker Corp., Billerica, MA, USA) with a horizontal bore magnet and a 72-mm birdcage coil. 2D tagged images were acquired at 10 frames per cardiac cycle using a FLASH-cine-tagging sequence with ECG and respiration triggering. The parameters were as follows: repetition time = 115 ms, echo time = 6 ms, flip angle = 20°, slice thickness = 0.8 mm, filter = distortion correction, phase oversampling = 10%, field of view = 150 mm, and matrix = 256 × 256. acquisition time for tagged MRI was 58 min and coronal 3D MRI was 30 min.

### 2.4. Motion Data Extraction from PET, 2D MRI, and 3D MRI

We analyzed the movement patterns and variations in movement during the respiratory cycle in the thoracic–abdominal region on both PET and MRI. Motion extraction was performed using a mutual information algorithm to register the mean image calculated from the whole image set after the first realignment. A 7-mm Gaussian kernel filter was applied to the PET images for smoothing prior to realignment. For the MRI, we used a 5-mm Gaussian kernel. Motion fields, which were used in motion correction, were estimated from the acquired PET image’s in vivo fiducial marker, 3D MRI, and 2D MRI. A 7th degree B-spline interpolation method was implemented to estimate the optimal transformation.

### 2.5. Motion Correction with PET, 2D MRI, and 3D MRI

We performed an image transformation for the motion correction of the PET data. An affine transformation was performed based on the matrix generated from the motion data [12]. The first frame of the gated PET image was set as the reference, and the other frames were co-registered to it using a transformation derived from the sieve images, 3D MRI, and 2D MRI. These transformations were used to correct the identical PET data in the image space.

A PET image acquired by measured optimal gate number was separated by each bin from the respiratory phase, and each frame of the image was adjusted by the coordinate information based on a mid-exhalation image and a rotation about an axis. A motion-corrected image was acquired from the sum of all transformed phase images.

### 2.6. PET Image Analysis

All the data were converted into standardized uptake value (SUV) for quantitative analysis [11]. The SUV PET image was visualized using trilinear interpolation. The sensitivity of the image was evaluated on count and signal-to-noise ratio (SNR) by drawn volume of interest (VOI). The VOI for measuring target count was spherical with a diameter of approximately 2 mm^3^, and was drawn on the target in the lung and liver region of each image. Background for calculating SNR and contrast was measured from VOI drawn on the surround of target region. SNR and contrast were calculated using the following equation: SNR = (Target count/Background standard deviation) × 100; Contrast = (Target count − Background)/Background.

The spatial resolution was evaluated on a full width at half maximum (FWHM) line profile. The line profile was drawn through the target region in both the horizontal and vertical directions on each image. The FWHMs were measured by Gaussian fitting from line profile. PET image analysis was performed using the Amide’s a medical image data examiner software. The results are presented as the mean ± SD (standard deviation).

### 2.7. Motion Compensation

We performed image transformation for motion correction of the PET data. Affine transformation was operated by matrix generated from motion data [24]. The acquired PET image by measured optimal gate number was separated by each bin from the respiratory phase. Each frame of the image was adjusted by the information of the coordinates, based on the mid-exhale image and the rotation of the axis. The motion-corrected image was acquired from the sum of all transformed phase images.

## 3. Results

### 3.1. Small Animal Molecular Sieve PET Imaging

**Figure 2:**
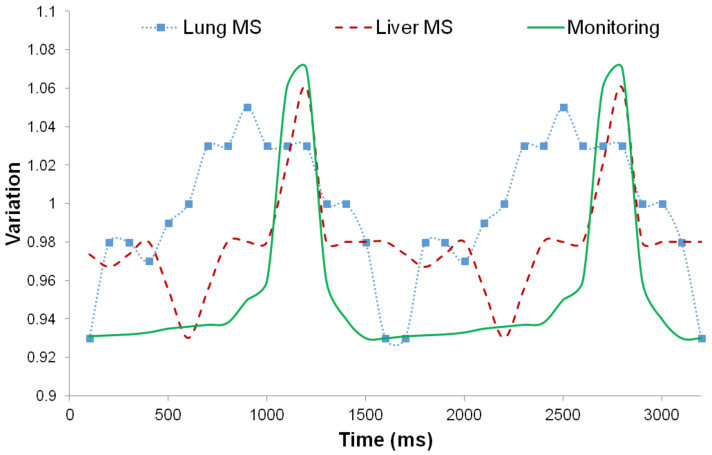
Results of detected motion variation in lung and liver during the PET image acquisition.

The horizontal and vertical length of the molecular sieve as motion target was 1.50 × 2.50 mm^2^. Figure 2 demonstrates the molecular sieve in each region in the obtained static and gated PET images. The SUV of molecular sieves in the high-activity group was more than 7, and in the low-activity group was less than 4. The horizontal and vertical FWHMs of the reference molecular sieve PET image were 1.43 mm and 2.91 mm, respectively.

The ECG and respiratory phases were simultaneously measured with PET image acquisition. ECG pulse signals of SD-Rats using three electrodes were approximately 200~350 bpm under 2% isoflurane anesthesia after surgery. Respiration signals of SD-Rats using a pneumatic sensor were approximately 25~40 bpm under 2% isoflurane anesthesia after surgery.

### 3.2. PET, 2D MRI, 3D MRI, and Tagged MRI

The horizontal and vertical lengths of the molecular sieve were 1.50 mm × 2.50 mm. The horizontal and vertical FWHM values of a reference molecular sieve image as a fiducial marker were 1.43 mm and 2.91 mm, respectively. Figure 1 presents images from static PET, gated PET, 3D VIBE MRI, 2D FLASH MRI, and 2D tagged MRI.

### 3.3. Motion Estimation from PET and MRI Images

Internal and external motion was measured in the lung region. The lung motion data are presented as they varied over the time interval in Figure 2. The internal motion data were measured from a gated PET image of a molecular sieve inserted into the body of a small animal, and the external motion data were measured from a molecular sieve adhered to the skin. Monitoring data measured with an external monitoring device are also presented in Figure 2. The motion data were normalized based on the monitoring data. The results showed that the internal variation in the lung region was on average 30–40% higher than the external variation. On the other hand, the results from the liver region revealed that the internal variation was lower than the external variation. In both regions of the small animals, the monitoring data were different from the internal motion measured directly from the molecular sieve.

**Figure 3:**
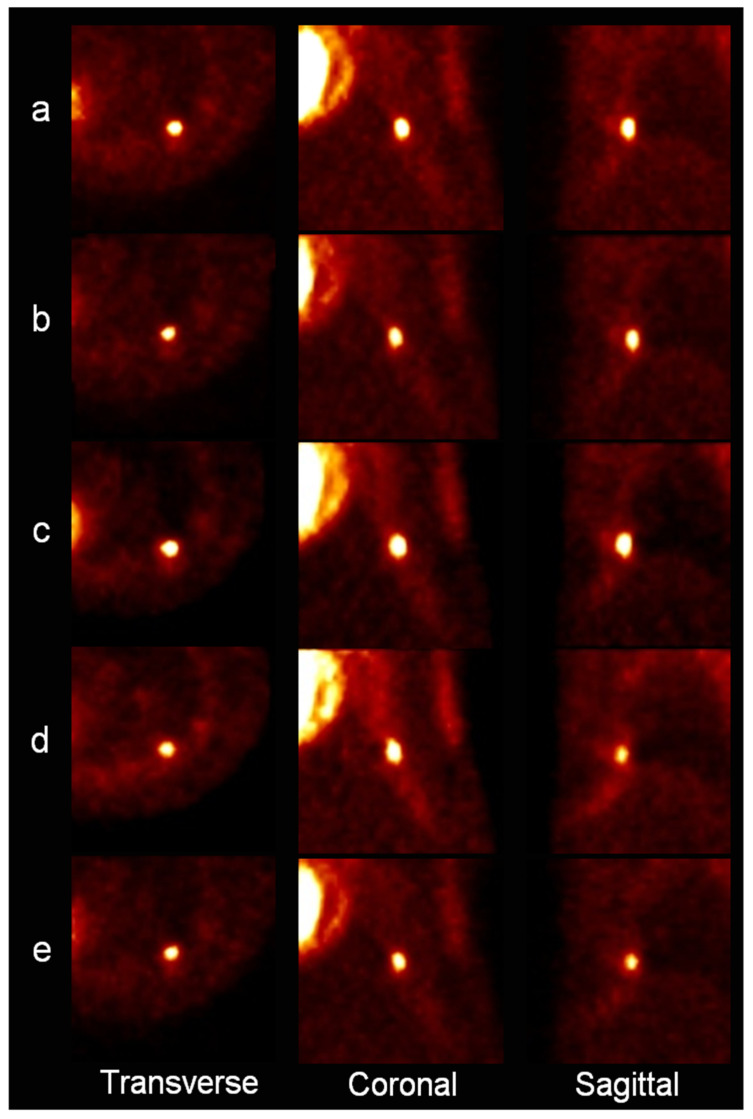
PET images of the molecular sieve in the lung region; (**a**) a static PET image, (**b**) a PET image corrected using a fiducial marker, (**c**) an image corrected using 3D VIBE MRI, (**d**) an image corrected using 2D FLASH MRI, (**e**) an image corrected using a 2D tagged MRI.

The estimated variations in the translations (X, Y, and Z axes) of the lung sieve in the PET image were 2.98, 0.71, and 1.42 mm, respectively. The estimated rotation degrees according to each axis were 0.02, 0.05, and 0.13, respectively. After motion correction using the sieve motion data, the estimated variation in the translations was 0.25, 0.29, and 0.32 mm, respectively. The estimated rotation degrees were 0.01, 0.01, and 0.01, respectively. After motion correction was performed by applying the motion data derived from the 3D MRI, the estimated variations in the translations in the lung PET image were 0.11, 0.08, and 0.21 mm, respectively. The estimated rotation degrees were 0.0051, 0.0012, and 0.0047, respectively. The estimated variations in translations in the lung region using a 2D MRI FLASH sequence were 2.6 and 1.3 mm for the X and Z axes, respectively. The estimated variations using a 2D tagged MRI were 1.9 and 2.3 mm for the X and Z axes, respectively. The results described above are shown as a graph in Figure 3.

### 3.4. Regional Motion Estimation

We measured internal motion in the thorax abdomen region. The lung and liver motion data are demonstrated through the variation with time intervals in Figure 3. Internal motion data were measured from inserted molecular sieve in small animal body of gated PET image. Monitoring data measured from external monitoring device are also presented in the graph. In both the regions of small animals, monitoring data were different from the internal motion measured directly from molecular sieve.

### 3.5. Count, SNR, Contrast and FWHM Assessment in PET Image

**Figure 4:**
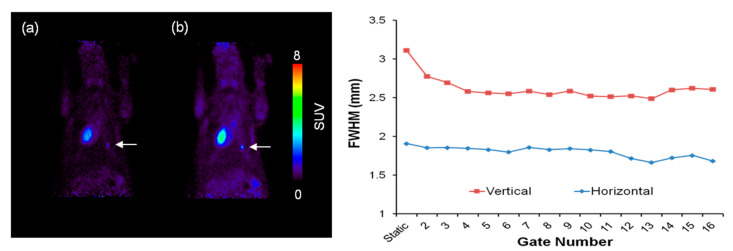
Motion correction PET image and result of FWHM in vertical and horizontal directions in the lung. (**a**) No motion correction PET image, (**b**) motion corrected PET image.

**Figure 5:**
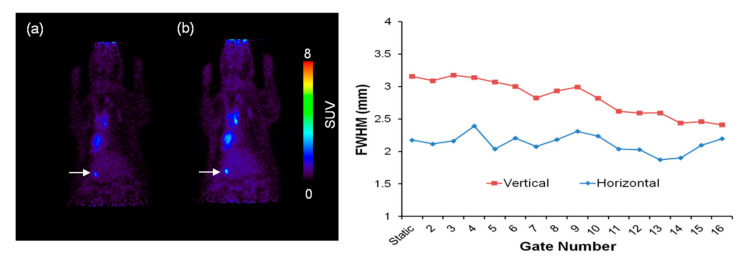
Motion correction PET image and result of FWHM in vertical and horizontal direction of liver. (**a**) No motion correction PET image, (**b**) motion corrected PET image.

We performed analysis of PET images by comparison of count, SNR, contrast and FWHM for determining gating effect regarding the organ region and tumor intensity. Figure 4 shows the estimated values of count, SNR and contrast in both the high-activity group and low-activity group in the lung region. Estimated counts (counts/s) in the lung target of the high-activity group were 9.31 ± 0.36, 8.12 ± 0.06, and 7.72 ± 0.09, and in the low-activity group were 3.49 ± 0.32, 3.57 ± 0.45, and 3.27 ± 0.52 for static, 4 bin and 8 bin images, respectively. Evaluated SNRs in the lung target of the high-activity group were 108.07 ± 11.01, 51.69 ± 2.90, and 28.79 ± 0.61, and in the low-activity group were 32.57 ± 1.44, 11.01 ± 2.87, and 8.96 ± 3.75 for the static, 4 bin and 8 bin images, respectively. Evaluated contrasts in the lung target of the high-activity group were 10.01, 8.36 and 6.97, and in the low-activity group were 3.57, 4.08 and 6.19, for static, 4 bin and 8 bin images, respectively (Table 1). Horizontal and vertical FWHMs in the lung target were 1.91 ± 0.17 and 3.11 ± 0.01 in the static image, 1.85 ± 0.22 and 2.58 ± 0.22 in the 4-bin image, and 1.83 ± 0.12 and 2.54 ± 0.18 in the 8 bin images (Figure 5a) (Table 2).

The result for the liver region exhibited a sharp decline in SNR and improvement in FWHM with the increase in gate number, as with the preceding result of the lung region. Estimated counts (counts/s) in the liver target of the high-activity group were 4.17 ± 0.07, 4.29 ± 0.26 and 4.18 ± 0.18, and in the low-activity group were 2.18 ± 0.06, 2.08 ± 0.08 and 2.13 ± 0.12 for static, 4 bin and 8 bin images, respectively. Evaluated SNR in the liver target of the high-activity group was 40.89 ± 4.89, 18.83 ± 0.50 and 13.52 ± 0.86, and in the low-activity group was 23.52 ± 4.40, 11.49 ± 1.02 and 7.96 ± 0.58 for static, 4 bin and 8 bin images, respectively (Table 3). Horizontal and vertical FWHMs were 2.18 ± 0.06 and 3.16 ± 0.13 in the static images, 2.39 ± 0.13 and 3.14 ± 0.00 in the 4 bin images, and 2.18 ± 0.07 and 2.94 ± 0.19 in the 8 bin images (Figure 5b). The liver region showed a higher slope of fitting line in the vertical FWHM graph (0.0553) when compared to the lung region (0.0177) (Table 4).

### 3.6. Motion Corrected PET Image

**Figure 6:**
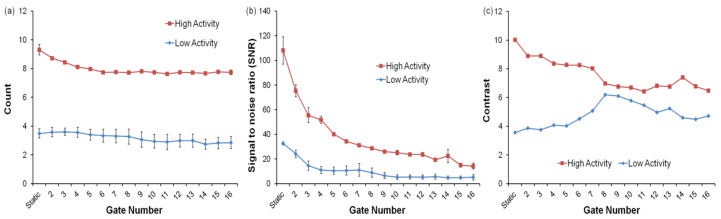
Gate number effects on the (**a**) count, (**b**) SNR, (**c**) contrast in high- and low-activity PET data.

**Figure 7:**
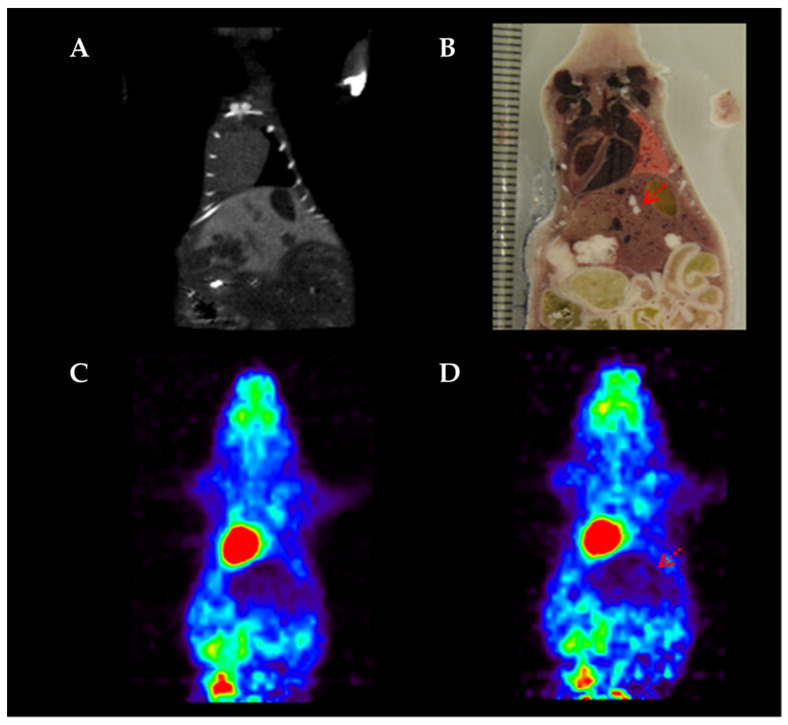
Detection of specific areas in liver. (**A**) Acquired CT data before PET scan, (**B**) Slice section of small animal, (**C**) high-activity PET image, (**D**) low-activity PET image.

Motion correction of small animal PET images was realized by affine transformation. Figure 6 presents images of an artificial tumor in lung region both before and after motion correction. Horizontal and vertical FWHMs were evaluated from each image for comparison, as shown in Table 5. In the lung region, horizontal and vertical FWHMs showed values of 2.04 and 2.63 before correction, and 1.89 and 2.11 after correction. Figure 7 also presents the image of an artificial tumor in the liver region both before and after motion correction. In the liver region, the previously mentioned criteria showed the values of 2.87 and 3.21 before correction, and 2.35 and 2.89 after correction.

## 4. Discussion

In the present work, we describe region-adaptive tumor evaluation by internal motion estimation for quantitative improvement of PET image [25]. It is hard to estimate real internal motion with conventional methods using external markers or external monitoring devices. Often, a surgical approach is necessary to determine tumor activity and internal motion during PET imaging [15]. Even though the fiducial mark is set by reference value, actual activity is difficult to measure due to the partial volume effect, the scatter effect or the attenuation effect [26]. Therefore, we tried quantitative estimation by surgical planting of artificial tumors of predetermined activity. The internal organ motion of small animals was measured from artificial lesions imitated by molecular sieves in the body. Surgery to insert artificial lesions containing radiopharmaceuticals in the lung and liver regions was performed after anterior thoracotomy. Suture, which followed the insertion, was appropriately performed, and we confirmed the absence of abnormal vital signs similar to preoperative conditions. The ^18^F adsorbed well into artificial tumors because of the immersion of the molecular sieves in ethanol before the initiation of study, for better enhancement of adsorptive power [27]. The Pluronic F-127 hydrogel that was used as coating material aided the artificial lesion to maintain activity while minimizing variation in the body. Therefore, the count of the target region was four times greater than the count of the organ region even after FDG was distributed completely over the entire body. The quantitative estimation was possible because this count ratio, which meant a high contrast, was indicated in the whole image.

The internal motion data were evaluated from inserted molecular sieves in the lung and liver region, respectively. Upon comparison of the obtained data with the monitoring data, the internal motion data were not identical to the monitoring data as presented in Figure 3. The inserted molecular sieves in the lung and liver regions showed different internal organ motion. Movement patterns of molecular sieves were different between each region. The internal motion in the lung region showed higher variation than the liver region. Monitoring signals presenting only total motion would be a problem for tumor quantification, because each region showed different motion according to the nearest organ.

Gated PET images showed that there was no proportional improvement in the count, SNR and FWHM according to increased number of gates. In fact, a drastic reduction in SNR was observed with increased gate numbers because of the increase in noise due to loss of count. The obtained result exhibited overall improvement in horizontal and vertical FWHM with an increase in gate number.

Molecular sieves were classified into two groups (high and low) based on the amount of activity, which meant artificial tumor intensity. Although SNR was reduced in both groups according to increase gate number, contrast was increased in low-activity groups from 2 bin to 8 bin (Figure 4). The effect of an increase in gate number on the high-activity group was no stronger than the low-activity group; on the contrary, contrast deteriorated continuously in the high-activity group. This result indicates that high gate numbers are not necessary in imaging for terminal tumors or high-intensity tumors, but are essential for low-intensity tumor quantification. Moreover, gating of low-intensity tumors could aid in evaluation of new radiopharmaceuticals, because low-activity substances are mostly used in research and development.

We also recognized that vertical FWHM was more influenced by gate number than horizontal FWHM, because of the movement patterns of thoracic abdomen organs. The movement of lung and liver caused by respiration and heartbeat created a difference between FWHM and actual size of molecular sieve.

The internal motion of lung and liver showed different patterns and variance. Therefore, the gate number for motion correction should be differently set, depending on the organ motion. The vertical FWHM of the liver region showed higher slope when compared to the lung region, from which we concluded that the impact of gating in the liver region was greater than in the lung region. As can be seen in Figure 4 and Figure 5, when the gate number is 4 bin, the result in the lung region shows that count and SNR were appropriately maintained, while improvement in FWHM was observed. However, there was no significant improvement in FWHM when gating more than 4 bin (4 bin: 17.09%, 8 bin: 18.39%). The result for the liver region in 8 bin shows that count and SNR were preserved, while FWHM was sufficiently improved. Gating with few gate numbers in the liver region revealed little change in FWHM improvement in contrast to the lung region (4 bin: 0.57%, 8 bin: 7.02%). This indicated that tumor imaging is needed for at least 8 bin gating in liver region.

Motion correction was executed by affine transformation, as shown in Figure 6. A significant difference was observed from the visible image, and we also confirmed improvement in images evaluated by FWHM. Table 5 presents the details of quantitative improvement in images of each region through motion correction. We ascertained that the motion correction using regional adaptive gate images resulted in better spatial resolution for all the regions. Our preclinical study proposed that in future, the internal motion estimation method could be applied to human clinical studies, which provide the possibility of motion-conjecture modeling without employing any external monitoring systems.

The limitation of this study is that it is difficult to directly observe the motion of internal organs, such as the liver. Molecular sieves were used for indirect observation of internal organs, but the molecular sieves did not settle well in internal organs and moved, making it difficult to observe the organ motion. It is necessary to develop a model that predicts the movement of other internal organs, as well as a breathing model, in order to overcome the limitations of motion correction. In this paper, we described regional adaptive PET image acquisition by internal motion estimation using artificial tumors. Our study demonstrated that a radioactive molecular sieve inserted into the body can be used as an artificial tumor for accurate estimation of internal organ motion. We confirmed the necessity of gating in low-intensity tumor quantification by carrying out a comparative analysis between high- and low-activity groups. Gating in low-intensity tumors could assist in the research of new radiopharmaceuticals. Estimated internal motion revealed differences in movement pattern and variation according to organ region in small animals. Based on this evidence, we could determine the optimal gate number and perform motion correction in accordance with the motion characteristics of each organ. The presented adaptive PET imaging technique based on tumor region allowed us to obtain the regional motion-corrected image, which resolved the disadvantage of gated PET. In consequence, we could quantitatively improve tumor evaluation by considering tumor region and tumor intensity.

In high-dose PET images, the targeted internal organs are clearly visible, but the background is also strongly visible. Because of this, it is difficult to analyze internal organ motion with a severe background, such as the liver. As shown in the results of this experiment, using low-dose PET showed that movement correction was possible not only in the lungs with a moderate background, but also in the liver with a severe background.

## Figures and Tables

**Table 1:** Estimated count (counts/s) and estimated SNR in lung.

Estimated count (counts/s) in lung		
	High-activity group	low-activity group
	9.31 ± 0.36	3.49 ± 0.32
	8.12 ± 0.06	3.57 ± 0.45
	7.72 ± 0.09	3.27 ± 0.52
Evaluated SNR in lung		
	High-activity group	low-activity group
	108.07 ± 11.01	32.57 ± 1.44
	51.69 ± 2.90	11.01 ± 2.87
	28.79 ± 0.61	8.96 ± 3.75

**Table 2:** Horizontal and vertical FWHM in lung.

Static Image	4 Bin Images	8 Bin Images
1.91 ± 0.17	1.85 ± 0.22	1.83 ± 0.12
3.11 ± 0.01	2.58 ± 0.22	2.54 ± 0.18

**Table 3:** Estimated count (counts/s) and estimated SNR.

Estimated count (counts/s) in liver		
	High-activity group	low-activity group
	4.17 ± 0.07	2.18 ± 0.06
	4.29 ± 0.26	2.08 ± 0.08
	4.18 ± 0.18	2.13 ± 0.12
Evaluated SNR in liver		
	High-activity group	low-activity group
	40.89 ± 4.89	23.52 ± 4.40
	18.83 ± 0.50	11.49 ± 1.02
	13.52 ± 0.86	7.96 ± 0.58

**Table 4:** Horizontal and vertical FWHM.

Static Image	4 Bin Images	8 Bin Images
2.18 ± 0.06	2.39 ± 0.13	2.18 ± 0.07
3.16 ± 0.13	3.14 ± 0.00	2.94 ± 0.19

**Table 5:** Horizontal and vertical FWHM values of an ^18^F-FDG PET image in the lung region.

FWHM	Uncorrected	PET Motion Correction (Fiducial)	Using 3D MRI Motion Correction (VIBE)	Using 2D MRI Motion Correction (FLASH)	Using 2D MRI Motion Correction (Tagging)
Horizontal	3.39 ± 0.08	3.31 ± 0.22	3.65 ± 0.05	2.99 ± 0.04	2.77 ± 0.06
Vertical	5.03 ± 0.11	4.54 ± 0.26	4.59 ± 0.06	4.51 ± 0.05	4.05 ± 0.08

## Data Availability

Data sharing not applicable.
